# Two polymorphs of 2-(prop-2-yn-1-yl­oxy)naphthalene-1,4-dione: solvent-dependent crystallization

**DOI:** 10.1107/S2056989018015438

**Published:** 2018-11-09

**Authors:** Flaviano Melo Ottoni, Raquel Geralda Isidório, Ricardo José Alves, Nivaldo Lúcio Speziali

**Affiliations:** a Departamento de Produtos Farmacêuticos, Faculdade de Farmácia, Universidade Federal de Minas Gerais, Avenida Antônio Carlos, 6627, Belo Horizonte, MG, CEP 31.270-901, Brazil; b Departamento de Física, Instituto de Ciências Exatas, Universidade Federal de Minas Gerais, Avenida Antônio Carlos, 6627, Belo Horizonte, MG, CEP 31.270-901, Brazil

**Keywords:** crystal structure, naphthalene-1,4-dione, naphtho­quinone, polymorphs

## Abstract

The title compound, C_13_H_8_O_3_, crystallizes in two polymorphs, namely the monoclinic (space group *P*2_1_/*c*) and triclinic (space group *P*ī) forms, obtained from *N*,*N*-di­methyl­formamide and isopropyl alcohol solutions, respectively. The mol­ecular structures and conformations in the two forms are essentially the same as each other. The naphtho­quinone ring systems are essentially planar with r.m.s. deviations of 0.015 and 0.029 Å for the monoclinic and triclinic forms, respectively. The *O*-propargyl groups are coplanar with the naphtho­quinone units with r.m.s deviations ranging from 0.04 to 0.09 Å. In the monoclinic crystal, mol­ecules are linked *via* pairs of C—H⋯O hydrogen bonds, forming a tape structure running along [120]. The tapes are further linked by a C—H⋯π inter­action into a layer parallel to the *ab* plane. Adjacent layers are linked by another C—H⋯π inter­action. In the triclinic crystal, mol­ecules are linked *via* C—H⋯O and π–π inter­actions, forming a layer parallel to the *ab* plane. Adjacent layers are linked by a C—H⋯π inter­action.

## Abstract 2

The title compound crystallizes in monoclinic (space group *P*2_1_/*c*) and triclinic (space group *P*ī) forms from *N*,*N*-di­methyl­formamide and isopropyl alcohol solutions, respectively. The planar structures of the mol­ecules in both crystals are essentially the same as each other, with maximum deviations of 0.0969 (11) and 0.209 (4) Å for the monoclinic and triclinic forms, respectively, from the mean planes of all non-H atoms.

## Chemical context   

Naphtho­quinone derivatives have been studied intensively over the past few decades, mostly because of their numerous biological activities, mainly anti­microbial and anti­tumor (Fujii *et al.*, 1992 ▸; Hussain *et al.*, 2007 ▸; Epifano *et al.*, 2014 ▸). The main mechanism of the activity is related to the formation of reactive oxygen species (ROS) through semiquinonic radicals, which cause damage to cell macromolecules and consequently cell death (Da Silva *et al.*, 2003 ▸). Among the substances that comprise this class, some synthetic bioactive derivatives have been obtained from lawsone (2-hy­droxy­naphthalene-1,4-dione) (Jordão *et al.*, 2015 ▸). In a basic medium, lawsone shows three sites able to be alkyl­ated (Lamoureux *et al.*, 2008 ▸), resulting in *O*-alkyl and *C*-alkyl derivatives difficult to purify in some cases in some cases (Kongkathip *et al.*, 2003 ▸). The title compound was obtained in higher yields since oxygen better accommodates the negative charge generated in the enolate formation, using a weak base, propargyl bromide, aprotic solvent and heat. The product has an alkyne terminal chain and can be used as the starting material in the synthesis of triazole derivatives, which are widely exploited in medicinal chemistry (Haider *et al.*, 2014 ▸). The present study shows that the title compound has two polymorphs, monoclinic (space group *P*2_1_/*c*) and triclinic (space group *P*ī), crystallized from *N*,*N*-di­methyl­formamide (DMF) and isopropyl alcohol, respectively.
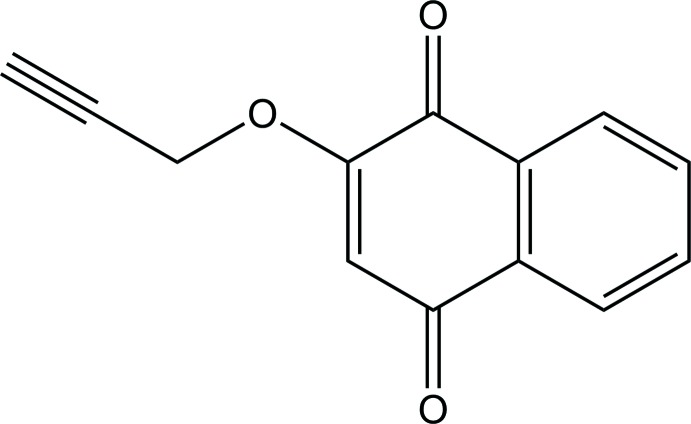


## Structural commentary   

**Figure 1:**
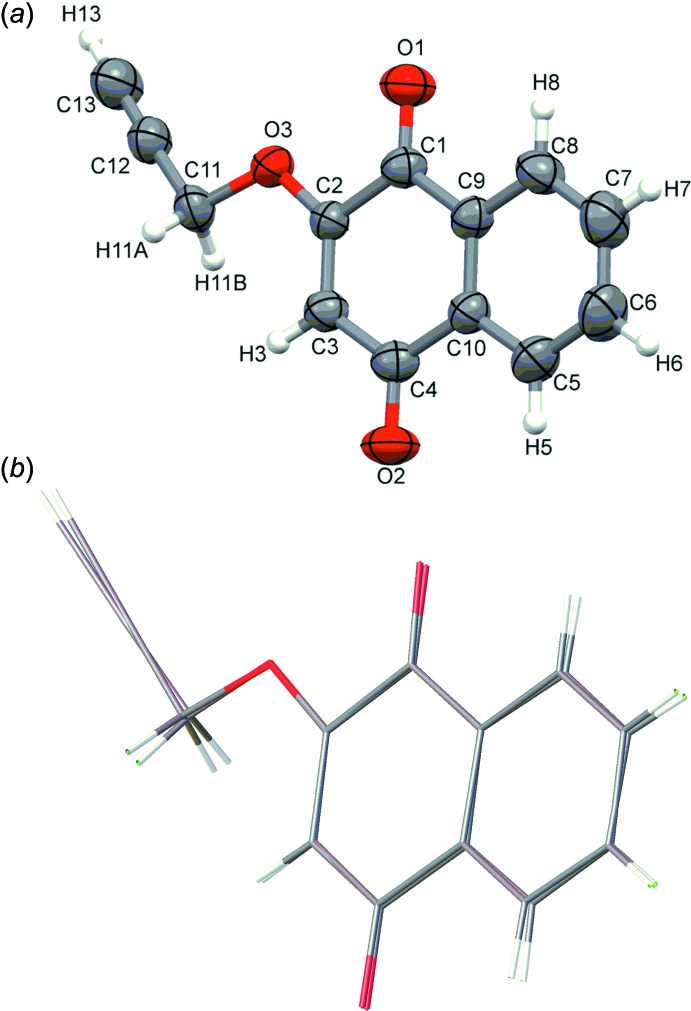
(*a*) The mol­ecular structure of the title compound (monoclinic form) with the atom labelling. Displacement ellipsoids of non-H atoms are drawn at the 50% probability level. (*b*) A view of the overlay of the mol­ecular structures of the monoclinic and triclinic forms of the title compound.

The mol­ecular structures in the two polymorphs are essentially the same (Fig. 1 ▸). The naphtho­quinone ring systems in the monoclinic and triclinic forms are both planar, with r.m.s. deviations of 0.015 and 0.029 Å, respectively, for the non-H atoms. Each propargyl group is coplanar with the naphtho­quinone ring system, with C1—C2—O3—C11 and C2—O3—C11—C12 torsion angles being −178.8 (1) and 175.9 (1)°, respectively, for the monoclinic form, and −177.1 (3) and −171.9 (3)°, respectively, for the triclinic form.

## Supra­molecular features   

**Figure 2:**
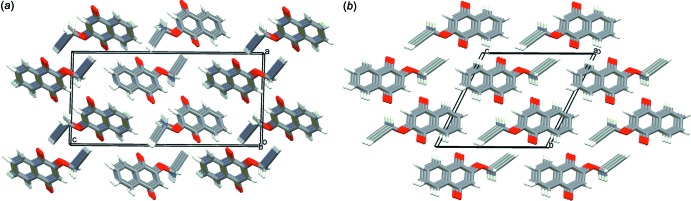
Packing diagrams of the title compound, showing the stacked naphtho­quinone mol­ecules: (*a*) monoclinic form viewed along the *b* axis and (*b*) triclinic form viewed along the *a* axis.

**Figure 3:**
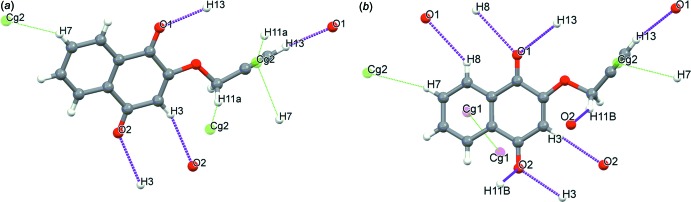
Selected inter­molecular inter­actions in the crystals of (*a*) the monoclinic form and (*b*) the triclinic form. Purple dashed lines represent the C—H⋯O hydrogen bonds and green dashed lines the C—H⋯π and π–π inter­actions. *Cg*1 is the centroid of the C5–C10 ring, while *Cg*2 is the midpoint of the C12≡C13 bond.

**Figure 4:**
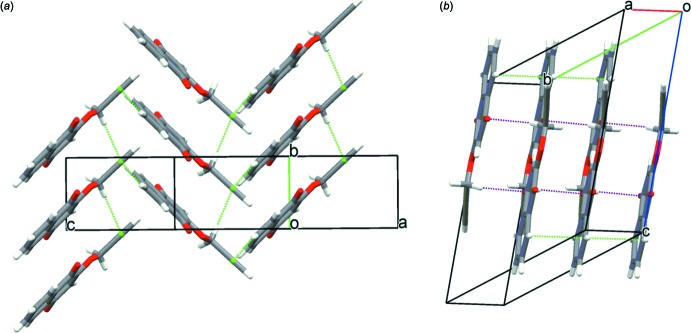
Partial packing diagrams of (*a*) the monoclinic form and (*b*) the triclinic from. Purple dashed lines represent the C—H⋯O hydrogen bonds and green dashed lines the C—H⋯π and π–π inter­actions.

The mol­ecular arrangements in both crystals are similar (Fig. 2 ▸) with nearly the same crystal densities (ρ = 1.383 and 1.392 Mg m^−3^ for the monoclinic and triclinic forms, respect­ively). In the monoclinic crystal, mol­ecules are linked *via* pairs of C—H⋯O hydrogen bonds (C3—H3⋯O2^i^ and C13—H13⋯O1^ii^; symmetry codes as in Table 1 ▸), forming a tape structure running along [120]. The tapes are further linked by a C—H⋯π inter­action (C11—H11*A*⋯*Cg*2^iii^; Table 1 ▸) into a layer parallel to the *ab* plane; *Cg*2 is the centroid of the C12≡C13 triple bond [Fig. 3 ▸(*a*)]. In the layer, mol­ecules are arranged parallel to each other and adjacent layers are linked by another C—H⋯π inter­action (C7—H7⋯*Cg*2^iv^; Table 1 ▸), forming a three-dimensional network [Fig. 4 ▸(*a*)]. In the tri­clinic crystal, mol­ecules are linked *via* C—H⋯O inter­actions (C3—H3⋯O2^i^, C11—H11*B*⋯O2 ^ii^ and C13—H13⋯O1^iii^; Table 1 ▸) and π–π inter­actions with centroid-centroid distances of 3.9906 (18) and 3.991 (2) Å, respectively, between C1–C4/C10/C9 rings and between C5–C10 rings, forming a layer parallel to the *ab* plane [Fig. 3 ▸(*b*)]. Adjacent layers are linked by a C—H⋯π inter­action [Fig. 4( ▸ *b*); C7—H7⋯*Cg*2^v^; Table 1 ▸].

## Database survey   

A search of the Cambridge Structural Database (Version 5.38; Groom *et al.*, 2016 ▸) for naphthalene-1,4-dione gave about 790 structures. Among them, 2-meth­oxy­naphthalene-1,4-dione (Jin *et al.*, 2011 ▸) and 2-{[1-(4-bromo­benz­yl)-1*H*-1,2,3-triazol-4-yl]meth­oxy}naphthalene-1,4-dione (Raja *et al.*, 2015 ▸) are very similar to the title compound. These compounds exhibit additional functional groups linked at O3 and essentially planar naphtho­quinone ring systems and C—H⋯O and π–π inter­actions are also observed in their crystal structures.

## Synthesis and crystallization   

The synthesis of the title compound was achieved in one step according to the literature method (Raja *et al.*, 2015 ▸). To a solution of lawsone (0.20 g, 1.15 mmol) in DMF (10 ml) was added K_2_CO_3_ (0.16 g, 1.15 mmol) and propargyl bromide (0.48 g, 4.07 mmol). The mixture was stirred at 363 K for 24 h. Then hydro­chloric acid (1.0 mol l^−1^, 0.34 ml) was added and the resulting solution was extracted with di­chloro­methane (3 × 25 ml). The organic layers were washed with water (60 ml), dried over anhydrous sodium sulfate and concentrated. The solid obtained was purified by column chromatography using silica gel and hexa­ne–ethyl acetate (9:1) and furnished the title compound in 70% yield. Yellow single crystals of the monoclinic and triclinic forms (m.p. 420.0–423.1 K) suitable for X-ray diffraction were obtained by slow evaporation of DMF and isopropyl alcohol solutions (about 0.5 mg ml-1), respectively, at room temperature.

Spectrometric data. IR ν_max_ (cm^−1^): The spectrum show the characteristic absorption bands of the main functional groups for title compound at IR (ν max/cm^−1^): 3250 (C—H alkyne), 3053 (C—H aromatic), 2130 (C≡C) 1649, 1680 (C=O quinone), 1575–1604 (C—C aromatic), 1016, 1208 and 1245 (C—O).^1^H NMR (400 MHz, CDCl_3_): *δ*_H_ 8.12 (*dd*, 1H, *J*_5,6_ 7.1 Hz, *J*_8,7_ 1.9 Hz, H-5), 8,07 (*dd*, 1H, *J*_8,7_ 7.0 Hz, *J*_8,6_ 1.9 Hz, H-8), 7.74 (*td*, 1H, *J*_6,5_ 7.5 Hz, *J*_6,7_ 7.5 Hz, *J*_6,8_ 1.7 Hz, H-6), 7.70 (*td*, 1H, *J*_7,6_ 7.4 Hz, *J*_7,8_ 7.4 Hz, *J*_7,5_ 1.6 Hz, H-7), 6.33 (*s*, 1H, H-3), 4.78 (*d*, 2H, *J*_11,13_ 2.4 Hz, H-11), 2.63 (*t*, 1H, *J*_13,11_ 2.4 Hz, H-13). ^13^C NMR (100 MHz, CDCl_3_): *δ*_C_ 184.6 (C-4), 179.8 (C-1), 158.1 (C-2), 134.3 (C-6), 133.4 (C-7), 131.9 (C-10), 131.1 (C-9), 126.7 (C-5), 126.2 (C-8), 111.6 (C-3), 78.2 (C-13), 75.5 (C-12), 56.7 (C-11).

## Refinement   

Crystal data, data collection and structure refinement details are summarized in Table 2 ▸. C-bound H atoms were constrained to an ideal geometry with C—H = 0.93–0.97 Å and with *U*_iso_(H) = 1.2*U*_eq_ (C).

## Supplementary Material

Crystal structure: contains datablock(s) Monoclinic, Triclinic, OPLAU. DOI: 10.1107/S2056989018015438/is5501sup1.cif

Supporting information file. DOI: 10.1107/S2056989018015438/is5501Monoclinicsup2.cml

CCDC references: 1876486, 1876485

Additional supporting information:  crystallographic information; 3D view; checkCIF report

## Figures and Tables

**Table 1:** Hydrogen-bond geometry (Å, °). *Cg*2 is the midpoint of the C12≡C13 bond.

*D*—H⋯A	*D*—H	H⋯*A*	*D*⋯*A*	*D*—H⋯*A*
**Monoclinic form**				
C3—H3⋯O2^i^	0.93	2.58	3.436 (2)	153
C13—H13⋯O1^ii^	0.93	2.33	3.350 (2)	173
C11—H11*A*⋯*Cg*2^iii^	0.97	2.91	3.740 (4)	145
C7—H7⋯*Cg*2^iv^	0.93	2.87	3.703 (4)	151
				
**Triclinic form**				
C3—H3⋯O2^i^	0.93	2.49	3.409 (4)	173
C11—H11*B*⋯O2^ii^	0.97	2.52	3.380 (4)	149
C13—H13⋯O1^iii^	0.93	2.44	3.340 (5)	164
C7—H7⋯*Cg*2^v^	0.93	2.93	3.829 (4)	162

Symmetry codes: monoclinic: (i) −1 − *x*, 2 − *y*, 1 − *z*; (ii) −*x*, −*y*, 1 − *z*; (iii) *x*, −1 + *y*, *z*; (iv) *x*, 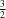 − *y*, −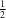 + *z*. Triclinic: (i) 2 − *x*, 1 − *y*, 1 − *z*; (ii) 1 − *x*, 1 − *y*, 1 − *z*; (iii) −*x*, 2 − *y*, 1 − *z*; (iv) −*x*, 2 − *y*, −*z*; (v) *x*, *y*, −1 + *z*.

**Table 2:** Experimental details

	Monoclinic	Triclinic
Crystal data		
Chemical formula	C_13_H_8_O_3_	C_13_H_8_O_3_
*M*_r_	212.19	212.19
Crystal system, space group	Monoclinic, *P*2_1_/*c*	Triclinic, *P* 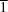
Temperature (K)	293	293
*a*, *b*, *c* (Å)	10.0911 (7), 4.8021 (3), 20.8939 (15)	3.9906 (6), 11.6943 (16), 12.3413 (16)
α, β, γ (°)	90, 91.174 (7), 90	63.347 (14), 83.343 (12), 83.018 (12)
*V* (Å^3^)	1012.27 (12)	509.69 (14)
*Z*	4	2
Radiation type	Mo *K*α	Mo *K*α
μ (mm^−1^)	0.10	0.10
Crystal size (mm)	0.35 × 0.2 × 0.1	0.35 × 0.2 × 0.1

Data collection		
Diffractometer	Rigaku Xcalibur Atlas Gemini ultra	Rigaku Xcalibur Atlas Gemini ultra
Absorption correction	Multi-scan (*CrysAlis PRO*; Rigaku OD, 2015 ▸)	Multi-scan (*CrysAlis PRO*; Rigaku OD, 2015 ▸)
*T*_min_, *T*_max_	0.764, 1.000	0.773, 1.000
No. of measured, independent and observed [*I* > 2σ(*I*)] reflections	10652, 3452, 1765	7730, 2503, 923
*R*_int_	0.058	0.084

Refinement		
*R*[*F* ^2^ > 2σ(*F* ^2^)], *wR*(*F* ^2^), *S*	0.059, 0.156, 1.01	0.064, 0.184, 1.00
No. of reflections	3452	2503
No. of parameters	145	145
H-atom treatment	H-atom parameters constrained	H-atom parameters constrained
Δρ_max_, Δρ_min_ (e Å^−3^)	0.19, −0.20	0.24, −0.24

Computer programs: *CrysAlis PRO* (Rigaku OD, 2015 ▸), *SHELXT* (Sheldrick, 2015*a* ▸), *SHELXL2014* (Sheldrick, 2015*b* ▸) and *OLEX2* (Dolomanov *et al.*, 2009 ▸).

## supplementary crystallographic information

### 2-(Prop-2-yn-1-yloxy)naphthalene-1,4-dione (Monoclinic) Crystal data

**Table:**

C_13_H_8_O_3_	*F*(000) = 440
*M**_r_* = 212.19	*D*_x_ = 1.392 Mg m^−^^3^
Monoclinic, *P*2_1_/*c*	Mo *K*α radiation, λ = 0.71073 Å
*a* = 10.0911 (7) Å	Cell parameters from 1655 reflections
*b* = 4.8021 (3) Å	θ = 3.9–30.6°
*c* = 20.8939 (15) Å	µ = 0.10 mm^−^^1^
β = 91.174 (7)°	*T* = 293 K
*V* = 1012.27 (12) Å^3^	Rod, yellow
*Z* = 4	0.35 × 0.2 × 0.1 mm

### 2-(Prop-2-yn-1-yloxy)naphthalene-1,4-dione (Monoclinic) Data collection

**Table:**

Rigaku Xcalibur Atlas Gemini ultra diffractometer	3452 independent reflections
Radiation source: fine-focus sealed X-ray tube, Enhance (Mo) X-ray Source	1765 reflections with *I* > 2σ(*I*)
Graphite monochromator	*R*_int_ = 0.058
Detector resolution: 10.4186 pixels mm^-1^	θ_max_ = 32.8°, θ_min_ = 2.8°
ω scans	*h* = −13→15
Absorption correction: multi-scan (CrysAlis PRO; Rigaku OD, 2015)	*k* = −7→7
*T*_min_ = 0.764, *T*_max_ = 1.000	*l* = −30→31
10652 measured reflections	

### 2-(Prop-2-yn-1-yloxy)naphthalene-1,4-dione (Monoclinic) Refinement

**Table:**

Refinement on *F*^2^	Primary atom site location: dual
Least-squares matrix: full	Hydrogen site location: inferred from neighbouring sites
*R*[*F*^2^ > 2σ(*F*^2^)] = 0.059	H-atom parameters constrained
*wR*(*F*^2^) = 0.156	*w* = 1/[σ^2^(*F*_o_^2^) + (0.0571*P*)^2^] where *P* = (*F*_o_^2^ + 2*F*_c_^2^)/3
*S* = 1.00	(Δ/σ)_max_ < 0.001
3452 reflections	Δρ_max_ = 0.19 e Å^−^^3^
145 parameters	Δρ_min_ = −0.20 e Å^−^^3^
0 restraints	

### 2-(Prop-2-yn-1-yloxy)naphthalene-1,4-dione (Monoclinic) Special details

**Table:**

Geometry. All esds (except the esd in the dihedral angle between two l.s. planes) are estimated using the full covariance matrix. The cell esds are taken into account individually in the estimation of esds in distances, angles and torsion angles; correlations between esds in cell parameters are only used when they are defined by crystal symmetry. An approximate (isotropic) treatment of cell esds is used for estimating esds involving l.s. planes.

### 2-(Prop-2-yn-1-yloxy)naphthalene-1,4-dione (Monoclinic) Fractional atomic coordinates and isotropic or equivalent isotropic displacement parameters (Å^2^)

**Table:**

	*x*	*y*	*z*	*U*_iso_*/*U*_eq_	
O1	0.08336 (12)	0.4803 (2)	0.34723 (5)	0.0512 (3)	
O2	0.50658 (13)	1.0905 (3)	0.41264 (6)	0.0646 (4)	
O3	0.19024 (11)	0.3978 (2)	0.46005 (5)	0.0493 (3)	
C1	0.17768 (15)	0.6268 (3)	0.36127 (7)	0.0381 (4)	
C2	0.24795 (16)	0.5949 (3)	0.42459 (7)	0.0390 (4)	
C3	0.35379 (16)	0.7492 (3)	0.44063 (7)	0.0434 (4)	
H3	0.3937	0.7248	0.4807	0.052*	
C4	0.40849 (16)	0.9546 (3)	0.39706 (8)	0.0419 (4)	
C5	0.38922 (16)	1.1922 (3)	0.29135 (8)	0.0445 (4)	
H5	0.4645	1.2953	0.3021	0.053*	
C6	0.32525 (17)	1.2340 (4)	0.23311 (8)	0.0495 (4)	
H6	0.3576	1.3651	0.2046	0.059*	
C7	0.21324 (18)	1.0817 (4)	0.21695 (8)	0.0528 (5)	
H7	0.1701	1.1116	0.1778	0.063*	
C8	0.16518 (16)	0.8854 (4)	0.25877 (8)	0.0460 (4)	
H8	0.0899	0.7832	0.2477	0.055*	
C9	0.22882 (15)	0.8402 (3)	0.31716 (7)	0.0362 (3)	
C10	0.34126 (15)	0.9961 (3)	0.33397 (7)	0.0363 (4)	
C11	0.24569 (19)	0.3468 (3)	0.52323 (7)	0.0488 (4)	
H11A	0.2477	0.5175	0.5480	0.059*	
H11B	0.3354	0.2761	0.5204	0.059*	
C12	0.16027 (19)	0.1408 (4)	0.55324 (8)	0.0510 (5)	
C13	0.0907 (2)	−0.0220 (4)	0.57682 (9)	0.0649 (6)	
H13	0.0352	−0.1518	0.5956	0.078*	

### 2-(Prop-2-yn-1-yloxy)naphthalene-1,4-dione (Monoclinic) Atomic displacement parameters (Å^2^)

**Table:**

	*U*^11^	*U*^22^	*U*^33^	*U*^12^	*U*^13^	*U*^23^
O1	0.0476 (7)	0.0589 (7)	0.0465 (7)	−0.0115 (6)	−0.0106 (5)	0.0087 (5)
O2	0.0593 (8)	0.0758 (9)	0.0578 (9)	−0.0230 (7)	−0.0195 (6)	0.0080 (7)
O3	0.0576 (8)	0.0521 (7)	0.0377 (6)	−0.0075 (6)	−0.0101 (5)	0.0117 (5)
C1	0.0386 (8)	0.0394 (8)	0.0362 (8)	0.0037 (7)	−0.0047 (6)	−0.0009 (7)
C2	0.0474 (9)	0.0381 (8)	0.0315 (8)	0.0030 (7)	−0.0020 (7)	0.0039 (6)
C3	0.0494 (9)	0.0474 (9)	0.0330 (8)	0.0008 (8)	−0.0112 (7)	0.0019 (7)
C4	0.0415 (9)	0.0438 (9)	0.0402 (9)	0.0014 (7)	−0.0068 (7)	−0.0023 (7)
C5	0.0397 (8)	0.0453 (9)	0.0485 (10)	−0.0006 (7)	0.0016 (7)	0.0030 (8)
C6	0.0524 (10)	0.0523 (10)	0.0439 (10)	0.0008 (8)	0.0037 (8)	0.0122 (8)
C7	0.0571 (11)	0.0630 (11)	0.0380 (9)	0.0015 (9)	−0.0095 (8)	0.0115 (8)
C8	0.0453 (9)	0.0519 (10)	0.0405 (9)	−0.0034 (8)	−0.0086 (7)	0.0065 (7)
C9	0.0367 (8)	0.0374 (8)	0.0343 (8)	0.0040 (7)	−0.0028 (6)	0.0017 (6)
C10	0.0361 (8)	0.0369 (8)	0.0356 (8)	0.0060 (6)	−0.0025 (6)	−0.0011 (6)
C11	0.0655 (11)	0.0482 (10)	0.0323 (8)	−0.0007 (9)	−0.0078 (7)	0.0043 (7)
C12	0.0674 (12)	0.0489 (10)	0.0366 (9)	0.0023 (9)	−0.0013 (8)	0.0006 (8)
C13	0.0780 (15)	0.0633 (12)	0.0536 (12)	−0.0049 (11)	0.0071 (10)	0.0095 (10)

### 2-(Prop-2-yn-1-yloxy)naphthalene-1,4-dione (Monoclinic) Geometric parameters (Å, º)

**Table:**

O1—C1	1.2145 (18)	C8—C7	1.380 (2)
O2—C4	1.2241 (18)	C8—H8	0.9300
O3—C2	1.3423 (18)	C9—C10	1.398 (2)
O3—C11	1.4442 (19)	C9—C1	1.478 (2)
C1—C2	1.496 (2)	C9—C8	1.384 (2)
C2—C3	1.337 (2)	C10—C4	1.483 (2)
C3—H3	0.9300	C10—C5	1.390 (2)
C4—C3	1.458 (2)	C11—C12	1.462 (3)
C5—C6	1.380 (2)	C11—H11A	0.9700
C5—H5	0.9300	C11—H11B	0.9700
C6—C7	1.382 (2)	C12—C13	1.167 (2)
C6—H6	0.9300	C13—H13	0.9300
C7—H7	0.9300		
			
O1—C1—C9	122.19 (14)	C6—C5—H5	120.0
O1—C1—C2	120.61 (14)	C6—C7—H7	119.9
O2—C4—C10	121.15 (15)	C7—C8—C9	120.16 (16)
O2—C4—C3	120.59 (15)	C7—C6—H6	119.9
O3—C2—C1	110.92 (14)	C7—C8—H8	119.9
O3—C11—H11A	110.4	C8—C9—C10	119.80 (14)
O3—C11—H11B	110.4	C8—C9—C1	119.77 (14)
O3—C11—C12	106.62 (14)	C8—C7—C6	120.20 (16)
C2—O3—C11	117.37 (13)	C8—C7—H7	119.9
C2—C3—C4	121.94 (14)	C9—C10—C4	120.35 (14)
C2—C3—H3	119.0	C9—C1—C2	117.20 (14)
C3—C2—O3	127.29 (15)	C9—C8—H8	119.9
C3—C2—C1	121.78 (14)	C10—C9—C1	120.42 (14)
C3—C4—C10	118.26 (14)	C10—C5—H5	120.0
C4—C3—H3	119.0	H11A—C11—H11B	108.6
C5—C6—H6	119.9	C12—C11—H11A	110.4
C5—C6—C7	120.24 (15)	C12—C11—H11B	110.4
C5—C10—C9	119.55 (14)	C12—C13—H13	180.0
C5—C10—C4	120.10 (15)	C13—C12—C11	179.1 (2)
C6—C5—C10	120.05 (16)		
			
O1—C1—C2—O3	−1.5 (2)	C8—C9—C1—C2	−177.9 (1)
O1—C1—C2—C3	178.5 (1)	C9—C1—C2—O3	179.1 (1)
O2—C4—C3—C2	−179.0 (2)	C9—C10—C4—O2	−179.6 (2)
O3—C2—C3—C4	178.9 (1)	C9—C10—C5—C6	0.6 (2)
C1—C9—C8—C7	−179.2 (2)	C9—C8—C7—C6	0.1 (3)
C1—C9—C10—C5	178.8 (1)	C9—C1—C2—C3	−1.0 (2)
C1—C9—C10—C4	−1.7 (2)	C9—C10—C4—C3	−0.3 (2)
C1—C2—C3—C4	−1.0 (2)	C10—C9—C1—O1	−177.1 (1)
C2—O3—C11—C12	175.9 (1)	C10—C5—C6—C7	0.1 (3)
C5—C10—C4—O2	−0.1 (2)	C10—C9—C8—C7	0.7 (2)
C5—C6—C7—C8	−0.5 (3)	C10—C9—C1—C2	2.3 (2)
C8—C9—C1—O1	2.7 (2)	C10—C4—C3—C2	1.7 (2)
C8—C9—C10—C5	−1.0 (2)	C11—O3—C2—C3	1.2 (2)
C8—C9—C10—C4	178.5 (1)	C11—O3—C2—C1	−178.8 (1)

### 2-(Prop-2-yn-1-yloxy)naphthalene-1,4-dione (Triclinic) Crystal data

**Table:**

C_13_H_8_O_3_	*Z* = 2
*M**_r_* = 212.19	*F*(000) = 220
Triclinic, *P*1	*D*_x_ = 1.383 Mg m^−^^3^
*a* = 3.9906 (6) Å	Mo *K*α radiation, λ = 0.71073 Å
*b* = 11.6943 (16) Å	Cell parameters from 796 reflections
*c* = 12.3413 (16) Å	θ = 3.2–22.3°
α = 63.347 (14)°	µ = 0.10 mm^−^^1^
β = 83.343 (12)°	*T* = 293 K
γ = 83.018 (12)°	Rod, yellow
*V* = 509.69 (14) Å^3^	0.35 × 0.2 × 0.1 mm

### 2-(Prop-2-yn-1-yloxy)naphthalene-1,4-dione (Triclinic) Data collection

**Table:**

Rigaku Xcalibur Atlas Gemini ultra diffractometer	2503 independent reflections
Radiation source: fine-focus sealed X-ray tube, Enhance (Mo) X-ray Source	923 reflections with *I* > 2σ(*I*)
Graphite monochromator	*R*_int_ = 0.084
Detector resolution: 10.4186 pixels mm^-1^	θ_max_ = 29.5°, θ_min_ = 3.2°
ω scans	*h* = −5→5
Absorption correction: multi-scan (CrysAlis PRO; Rigaku OD, 2015)	*k* = −14→15
*T*_min_ = 0.773, *T*_max_ = 1.000	*l* = −16→16
7730 measured reflections	

### 2-(Prop-2-yn-1-yloxy)naphthalene-1,4-dione (Triclinic) Refinement

**Table:**

Refinement on *F*^2^	Primary atom site location: dual
Least-squares matrix: full	Hydrogen site location: inferred from neighbouring sites
*R*[*F*^2^ > 2σ(*F*^2^)] = 0.064	H-atom parameters constrained
*wR*(*F*^2^) = 0.184	*w* = 1/[σ^2^(*F*_o_^2^) + (0.0469*P*)^2^] where *P* = (*F*_o_^2^ + 2*F*_c_^2^)/3
*S* = 1.00	(Δ/σ)_max_ < 0.001
2503 reflections	Δρ_max_ = 0.24 e Å^−^^3^
145 parameters	Δρ_min_ = −0.23 e Å^−^^3^
0 restraints	

### 2-(Prop-2-yn-1-yloxy)naphthalene-1,4-dione (Triclinic) Special details

**Table:**

Geometry. All esds (except the esd in the dihedral angle between two l.s. planes) are estimated using the full covariance matrix. The cell esds are taken into account individually in the estimation of esds in distances, angles and torsion angles; correlations between esds in cell parameters are only used when they are defined by crystal symmetry. An approximate (isotropic) treatment of cell esds is used for estimating esds involving l.s. planes.

### 2-(Prop-2-yn-1-yloxy)naphthalene-1,4-dione (Triclinic) Fractional atomic coordinates and isotropic or equivalent isotropic displacement parameters (Å^2^)

**Table:**

	*x*	*y*	*z*	*U*_iso_*/*U*_eq_	
O1	0.1734 (6)	0.9294 (2)	0.1987 (2)	0.0641 (7)	
O2	0.9527 (6)	0.4894 (2)	0.3422 (2)	0.0669 (8)	
O3	0.3732 (5)	0.82577 (18)	0.41940 (19)	0.0502 (6)	
C1	0.3597 (7)	0.8310 (3)	0.2279 (3)	0.0434 (8)	
C2	0.4859 (7)	0.7629 (3)	0.3529 (3)	0.0403 (8)	
C3	0.6822 (7)	0.6529 (3)	0.3868 (3)	0.0446 (8)	
H3	0.7612	0.6142	0.4642	0.053*	
C4	0.7756 (7)	0.5920 (3)	0.3068 (3)	0.0469 (8)	
C5	0.7466 (8)	0.6005 (3)	0.1040 (3)	0.0623 (10)	
H5	0.8690	0.5209	0.1307	0.075*	
C6	0.6507 (9)	0.6636 (4)	−0.0147 (4)	0.0734 (12)	
H6	0.7105	0.6264	−0.0676	0.088*	
C7	0.4685 (9)	0.7804 (4)	−0.0543 (3)	0.0693 (11)	
H7	0.4083	0.8226	−0.1343	0.083*	
C8	0.3734 (8)	0.8360 (3)	0.0234 (3)	0.0544 (9)	
H8	0.2467	0.9147	−0.0038	0.065*	
C9	0.4678 (7)	0.7737 (3)	0.1427 (3)	0.0428 (8)	
C10	0.6598 (7)	0.6563 (3)	0.1823 (3)	0.0447 (8)	
C11	0.4891 (8)	0.7741 (3)	0.5405 (3)	0.0532 (9)	
H11A	0.7294	0.7831	0.5362	0.064*	
H11B	0.4510	0.6837	0.5849	0.064*	
C12	0.2992 (8)	0.8455 (3)	0.6013 (3)	0.0518 (9)	
C13	0.1377 (9)	0.9034 (3)	0.6476 (3)	0.0692 (11)	
H13	0.0087	0.9496	0.6846	0.083*	

### 2-(Prop-2-yn-1-yloxy)naphthalene-1,4-dione (Triclinic) Atomic displacement parameters (Å^2^)

**Table:**

	*U*^11^	*U*^22^	*U*^33^	*U*^12^	*U*^13^	*U*^23^
O1	0.0807 (17)	0.0553 (14)	0.0518 (16)	0.0214 (13)	−0.0130 (12)	−0.0243 (12)
O2	0.0784 (17)	0.0510 (14)	0.0670 (17)	0.0249 (13)	−0.0154 (13)	−0.0273 (12)
O3	0.0621 (14)	0.0495 (13)	0.0460 (14)	0.0123 (10)	−0.0128 (11)	−0.0294 (11)
C1	0.0431 (18)	0.0423 (18)	0.044 (2)	0.0002 (14)	0.0003 (15)	−0.0204 (16)
C2	0.0425 (18)	0.0415 (18)	0.040 (2)	0.0020 (14)	−0.0068 (14)	−0.0206 (16)
C3	0.0479 (19)	0.0440 (18)	0.0411 (19)	0.0029 (15)	−0.0038 (14)	−0.0195 (15)
C4	0.0456 (19)	0.0450 (19)	0.048 (2)	−0.0002 (15)	−0.0002 (15)	−0.0204 (16)
C5	0.068 (2)	0.068 (2)	0.064 (3)	0.0107 (19)	−0.0070 (19)	−0.044 (2)
C6	0.077 (3)	0.095 (3)	0.069 (3)	0.009 (2)	−0.008 (2)	−0.057 (3)
C7	0.073 (3)	0.089 (3)	0.052 (2)	0.003 (2)	−0.0072 (19)	−0.038 (2)
C8	0.059 (2)	0.055 (2)	0.043 (2)	0.0090 (17)	−0.0107 (17)	−0.0171 (18)
C9	0.0392 (18)	0.0492 (19)	0.045 (2)	−0.0027 (14)	0.0019 (15)	−0.0268 (16)
C10	0.0482 (19)	0.0462 (19)	0.043 (2)	0.0013 (15)	−0.0035 (15)	−0.0238 (16)
C11	0.051 (2)	0.066 (2)	0.050 (2)	0.0075 (17)	−0.0159 (17)	−0.0329 (19)
C12	0.056 (2)	0.058 (2)	0.046 (2)	0.0043 (17)	−0.0090 (17)	−0.0274 (18)
C13	0.078 (3)	0.079 (3)	0.054 (2)	0.018 (2)	−0.0136 (19)	−0.036 (2)

### 2-(Prop-2-yn-1-yloxy)naphthalene-1,4-dione (Triclinic) Geometric parameters (Å, º)

**Table:**

O1—C1	1.220 (3)	C7—H7	0.9300
O2—C4	1.236 (3)	C8—C7	1.378 (4)
O3—C2	1.339 (3)	C8—H8	0.9300
O3—C11	1.447 (3)	C9—C8	1.394 (4)
C1—C2	1.499 (4)	C10—C9	1.393 (4)
C1—C9	1.481 (4)	C10—C4	1.478 (4)
C2—C3	1.339 (3)	C10—C5	1.382 (4)
C3—H3	0.9300	C11—H11A	0.9700
C4—C3	1.449 (4)	C11—H11B	0.9700
C5—H5	0.9300	C12—C11	1.454 (4)
C5—C6	1.389 (5)	C12—C13	1.164 (4)
C6—H6	0.9300	C13—H13	0.9300
C6—C7	1.369 (4)		
			
O1—C1—C2	120.7 (3)	C6—C5—H5	120.1
O1—C1—C9	121.8 (3)	C7—C8—C9	119.7 (3)
O3—C2—C1	111.2 (2)	C7—C8—H8	120.1
O3—C11—C12	107.6 (2)	C7—C6—C5	120.2 (3)
O3—C11—H11A	110.2	C7—C6—H6	119.9
O3—C11—H11B	110.2	C8—C7—H7	119.7
O2—C4—C10	121.0 (3)	C8—C9—C1	120.2 (3)
O2—C4—C3	120.2 (3)	C9—C8—H8	120.1
C2—C3—C4	122.2 (3)	C9—C10—C4	120.3 (3)
C2—O3—C11	117.2 (2)	C9—C1—C2	117.5 (3)
C2—C3—H3	118.9	C10—C9—C1	120.1 (3)
C3—C2—O3	127.8 (3)	C10—C9—C8	119.7 (3)
C3—C2—C1	121.0 (3)	C10—C5—H5	120.1
C3—C4—C10	118.7 (3)	C10—C5—C6	119.9 (3)
C4—C3—H3	118.9	H11A—C11—H11B	108.5
C5—C10—C9	119.8 (3)	C12—C11—H11A	110.2
C5—C10—C4	119.9 (3)	C12—C11—H11B	110.2
C5—C6—H6	119.9	C12—C13—H13	180.0
C6—C7—C8	120.6 (4)	C13—C12—C11	177.6 (3)
C6—C7—H7	119.7		
			
O1—C1—C2—O3	−1.3 (4)	C5—C10—C9—C1	177.0 (3)
O1—C1—C2—C3	178.0 (3)	C5—C10—C9—C8	−2.0 (5)
O1—C1—C9—C10	−175.2 (3)	C5—C10—C4—O2	0.8 (5)
O1—C1—C9—C8	3.8 (5)	C5—C10—C4—C3	179.7 (3)
O2—C4—C3—C2	−178.8 (3)	C5—C6—C7—C8	−1.0 (6)
O3—C2—C3—C4	177.5 (3)	C9—C1—C2—O3	179.1 (3)
C1—C2—C3—C4	−1.7 (5)	C9—C1—C2—C3	−1.5 (5)
C1—C9—C8—C7	−178.5 (3)	C9—C10—C5—C6	1.9 (5)
C2—O3—C11—C12	−171.9 (3)	C9—C8—C7—C6	1.0 (6)
C2—C1—C9—C10	4.4 (5)	C10—C9—C8—C7	0.5 (5)
C2—C1—C9—C8	−176.6 (3)	C10—C4—C3—C2	2.3 (5)
C4—C10—C9—C1	−3.9 (5)	C10—C5—C6—C7	−0.5 (6)
C4—C10—C9—C8	177.1 (3)	C11—O3—C2—C1	−177.1 (3)
C4—C10—C5—C6	−177.1 (3)	C11—O3—C2—C3	3.7 (5)
